# Publication Bias in Laboratory Animal Research: A Survey on Magnitude, Drivers, Consequences and Potential Solutions

**DOI:** 10.1371/journal.pone.0043404

**Published:** 2012-09-05

**Authors:** Gerben ter Riet, Daniel A. Korevaar, Marlies Leenaars, Peter J. Sterk, Cornelis J. F. Van Noorden, Lex M. Bouter, René Lutter, Ronald P. Oude Elferink, Lotty Hooft

**Affiliations:** 1 Department of General Practice, Academic Medical Center, University of Amsterdam, Amsterdam, The Netherlands; 2 3R Research Centre, Central Animal Laboratory, Radboud University Nijmegen Medical Centre, Nijmegen, The Netherlands; 3 Department of Respiratory Medicine, Academic Medical Center, University of Amsterdam, Amsterdam, The Netherlands; 4 Department of Cell Biology and Histology, Academic Medical Center, University of Amsterdam, Amsterdam, The Netherlands; 5 Executive Board, VU University, Amsterdam, The Netherlands; 6 Tytgat Institute for Liver and Intestinal Research Academic Medical Center, University of Amsterdam, Amsterdam, The Netherlands; 7 Dutch Cochrane Centre, Academic Medical Center, University of Amsterdam, Amsterdam, The Netherlands; IUMSP, University Hospital Lausanne, Switzerland.

## Abstract

**Context:**

Publication bias jeopardizes evidence-based medicine, mainly through biased literature syntheses. Publication bias may also affect laboratory animal research, but evidence is scarce.

**Objectives:**

To assess the opinion of laboratory animal researchers on the magnitude, drivers, consequences and potential solutions for publication bias. And to explore the impact of size of the animals used, seniority of the respondent, working in a for-profit organization and type of research (fundamental, pre-clinical, or both) on those opinions.

**Design:**

Internet-based survey.

**Setting:**

All animal laboratories in The Netherlands.

**Participants:**

Laboratory animal researchers.

**Main Outcome Measure(s):**

Median (interquartile ranges) strengths of beliefs on 5 and 10-point scales (1: totally unimportant to 5 or 10: extremely important).

**Results:**

Overall, 454 researchers participated. They considered publication bias a problem in animal research (7 (5 to 8)) and thought that about 50% (32–70) of animal experiments are published. Employees (n = 21) of for-profit organizations estimated that 10% (5 to 50) are published. Lack of statistical significance (4 (4 to 5)), technical problems (4 (3 to 4)), supervisors (4 (3 to 5)) and peer reviewers (4 (3 to 5)) were considered important reasons for non-publication (all on 5-point scales). Respondents thought that mandatory publication of study protocols and results, or the reasons why no results were obtained, may increase scientific progress but expected increased bureaucracy. These opinions did not depend on size of the animal used, seniority of the respondent or type of research.

**Conclusions:**

Non-publication of “negative” results appears to be prevalent in laboratory animal research. If statistical significance is indeed a main driver of publication, the collective literature on animal experimentation will be biased. This will impede the performance of valid literature syntheses. Effective, yet efficient systems should be explored to counteract selective reporting of laboratory animal research.

## Introduction

Publication bias jeopardizes evidence-based medicine through biased literature syntheses of clinical studies. [1], [2] It is conceivable that non-publication practices affect laboratory animal research too.[3]–[6] In particular, non-reporting of “negative” research findings may hamper progress in laboratory animal research (LAR) through unnecessary duplications of experiments and may lead to premature first-in-man studies. Data on the extent of non-publication in LAR is scarce.[7]–[10] Historically, the outlook on publishing may be different between clinical and laboratory animal research. For example, in his book ‘Introduction à l'étude de la medicine experimentale’, the founding father of experimental physiology, Claude Bernard, argued that “*[.] in physiology we must never make average descriptions of experiments because the true relations of phenomena disappear in the average; [.] we must [.] present our most perfect experiment as a type”*. [11] More recently, Lemon and Dunnett, arguing against the use of systematic reviews for LAR, wrote that *“no mechanism exists for so called negative results to be published. [.]. This is not just an issue of publication bias [.]. Scientific experiments are designed to test for evidence in favour of a particular experimental hypothesis and to abandon it if insufficient evidence is acquired.”* [12] Against this background, we assessed laboratory animal researchers’ opinions about magnitude, drivers, consequences and potential solutions for publication bias in The Netherlands (575,278 animals used in experiments in 2010) [13]. We explored the impact of animal size, researcher seniority, working in a for-profit organization and type of research on those opinions.

## Methods

We approached respondents via a two-step procedure: (i) the Dutch professional association of animal welfare officers, and (ii) all animal welfare officers. In March 2011, we sent a standard letter of invitation to participate by email to all animal welfare officers in the Netherlands via one liaison person (ML) who had access to them through their professional association. Since the animal welfare officers have address lists of all researchers that (had) performed LAR in their institutes, they were asked to send the invitation letter to all their animal researchers. The invitation letters () contained the link to the internet-based survey and explained that confidentiality was guaranteed for the respondent and their institute. In the months prior to the survey’s launch, two authors (GtR and LH) had secured informal commitment to the survey from animal welfare officers in ten institutes.

The survey (Appendix S2) addressed five background features: (i) field of expertise, (ii) affiliation, (iii) size of the animals used [small (e.g. birds, rodents, fish, amphibians, reptiles) versus large (otherwise) or both], (iv) seniority of the respondent, as expressed by the number of (co-)authored publications, and (v) the type of the research of the respondent, where we defined pre-clinical research as LAR to investigate if a drug, procedure or treatment may have an effect in humans; other research was deemed basic. These background variables were also used in one-way stratified analyses to assess if results varied by these variables. The estimates for the publication rates were analyzed using bootstrapped quantile regression (200 repetitions) to adjust for the simultaneous effects of the four stratification variables. Items were scored on 5 or 10 point scales, in which 1 indicated totally unimportant and 5 or 10 extremely important.

We used Surveymonkey software (www.surveymonkey.com) and STATA software (version 10.1). Mann-Whitney and Kruskal-Wallis tests (in combination with Tukey’s post-hoc test) were used to assess statistical significance (alpha = 0.05). We applied the Bonferroni correction to adjust for multiple testing.

## Results

Through the Dutch professional association of animal welfare officers, all animal welfare officers (n = 39) received the invitation letter and an internet link for the survey. We estimate that between 2,000 and 3,500 laboratory animal researchers received the invitation to participate. Between 17 March and 30 July 2011, 474 (between 14–24%) laboratory animal researchers returned the survey. Fifty-one respondents did not fill in the survey completely. Of these, we excluded 20 because of absence of (at least) their background data. This left 454 participants for the analysis. The variation in the number of respondents across table 1 is caused by the other 31 respondents.

**Table 1:** Summary responses to a survey among Dutch experimental animal researchers.

Question†	Scale	N¶	Median (p25–p75)
6. Overall, what % of ethics-approved experiments performed in experimental animalresearch is published?	0–100%	454	50 (32–70)
7. Overall, what % of animal experiments you have been involved in have been published on?	0–100%	215§	80 (60–90)
8. Do you consider publication bias a problem for experimental animal research?	1–10 (not at all - extremely)	448	7 (5–8)
9. What are important causes of non-publication in experimental animal research?	1–5 (totally unimportant - very important)	444	
1. Lack of statistically significant differences (“negative” findings)			4 (4–5)
2. Instrumentation/technical problems			4 (3–4)
3. Lack of time to write manuscripts			2 (2–3)
4. Loss of interest			2.5 (2–3)
5. Many studies are seen as pilot studies only			3 (3–4)
10. Who are responsible for non-publication in experimental animal research?	1–5 (least important - most important)	440	
1. Senior researchers (supervisors)			4 (3–5)
2. Junior researchers (research fellow/PhD)			3 (2–4)
3. Editors			4 (3–4)
4. Reviewers/Referees			4 (3–5)
5. Funders			2 (1–4)
11. Publication bias is important for experimental animal research with respect to:	1–10 (totally unimportant – extremely important)	434	
1. Duplication of research efforts			8 (7–9)
2. Bias in literature reviews or meta-analyses			8 (7–9)
3. Initiation of phase-1 clinical trials in humans			7 (6–8)
12. Would you use initiatives to make the publishing of negative results or commentson why an experiment could not be completed much easier, for example an (anonymous)online database or (online) journals of negative result?	1–3 (1 = never, 2 = sometimes, 3 = often/always)	432	2 (2–3)
14. Mandatory anonymous publication of research protocols of all ethics-approved animalresearch experiments in a publically available database would change:	1–5 (extreme increase - extreme decrease)	426	
1. Duplication of research efforts			4 (3–4)
2. Validity of literature reviews			2 (2–3)
3. Certainty that competing investigators do not catch up			3 (3–4)
4. Bureaucracy			2 (1–3)
5. Overall scientific progress			2 (2–3)
**Question**	**Scale**	**N**	**Median (p25–p75)**
15. Mandatory anonymous publication of a brief structured form in a publically availabledatabase, that gave main results or explained why an experiment could not be completedwould change:	1–5 (extreme increase - extreme decrease)	423	
1. Duplication of research efforts			4 (3–4)
2. Validity of literature reviews			2 (2–3)
3. Certainty that competing investigators do not catch up			3 (3–4)
4. Bureaucracy			2 (2–3)
5. Overall scientific progress			2 (2–3)

† Questions are numbered according to the survey in which questions 1 to 5 enquired after respondents’ background characteristics (Appendix S2).

¶ Between 17 March and 30 July 2011, 474 laboratory animal researchers returned the survey. Fifty-one respondents did not fill in the survey completely. Of these, we excluded 20 because of absence of (at least) their background data. The variation in the total number of 454 respondents across table 1 is caused by the other 31 respondents.

§ The group that (co-)authored 0–5 studies was excluded from this row because very junior investigators very often had either zero or 100 percent of their papers publish.

Table 1 shows the main results. Table S1 shows the results stratified by four background variables. On average, those working in not-for-profit institutes (n = 421) estimated that 50 percent (interquartile range (IQR) 35 to 70) of all conducted laboratory animal experiments are published. Researchers in a for-profit environment (n = 21) estimated that only 10 percent (5 to 50) is published. Researchers in not-for-profit institutes reported that 80 percent (60 to 90) of their own work had been published against 10 percent (5 to 39) of the work of researchers in a for-profit environment. Respondents working only with large animals thought that their own work was published in 90 percent of cases (79 to 100) against 75 percent (50 to 90) for those working with small animals only. Results from the multivariable analyses change these results only slightly (Tables S2 and S3). In particular, respondents who had co-authored more than 5 papers estimated publication rates 10 percentage points higher than respondents who had published less (95% CI from 0.8 to 19.1). Respondents working with large animals only estimated the publication rate of work they had been involved in personally 10 percentage points higher (95% CI from 1.1 to 18.9). Statistical non-significance and technical problems are considered to be the main drivers for non-publication. Supervisors, editors, and reviewers were considered responsible for non-publication. As expected, funders were considered more important in a for-profit environment (2 versus 4). Overall, respondents considered publication bias an important problem for LAR (7 (IQR 5–8)) and for research duplication, literature syntheses and well-timed initiation of phase-1clinical trials in humans in particular. Table 1 shows that respondents thought mandatory publication of study protocols or results may help avoid unnecessary duplication, increase validity of literature syntheses and scientific progress, but at the cost of increased bureaucracy. These opinions did not depend on size of the animal used, seniority of the respondent or type of research.

## Discussion

Publication bias is an important problem in laboratory animal research (LAR) according to laboratory animal researchers. We estimate that only fifty percent of LAR is published, but it may be far less in for-profit organizations given that their employees estimated that only ten percent of LAR gets published overall, including their own. Lack of statistical significance, technical problems, the opinions of supervisors and peer reviewers were considered important drivers of non-publication. Respondents thought that mandatory publication of study protocols, research results or the reasons why results could not be obtained may accelerate scientific progress.

To our knowledge, this is the first survey among laboratory animal researchers focusing on publication bias. This survey has several limitations. First, we estimate the response rate to this survey to be between 14 and 24 percent. We do not know to which extent the results are representative for the Dutch LAR community, let alone for the wider LAR community. The number of laboratory animal researchers in The Netherlands is unknown. We were unable to obtain exact information from the institutes on to the number of E-mail addresses to which the survey had been sent. Another difficulty is that such address lists may not always be fully up to date. In particular, researchers who retire or change jobs may be listed in error. Second, the survey was restricted to one country. Third, only few researchers in for-profit organizations participated. Fourth, our results are reminiscent of the joke about surveys on driving ability in which 90% of respondents think that they belong to the group of people whose driving abilities are above-average. Likewise, it seems somewhat paradoxical that our respondents estimate the publication rate of their own work as much higher (in theory, they could have calculated it) than the overall rate. Another explanation may be that the 50% rate mentioned in the introduction to the survey acted as an anchor that made respondents estimate the overall rates as too low. That would imply that a non-publication rate of 20% is closer to the truth. This issue is related to the next one. Fifth, our study investigated researchers’ opinions, which may not reflect the true rate(s) of non-publication. Sixth, due to the large number of statistical significance tests (n = 121), application of the Bonferroni correction for multiple testing (at alpha = 0.05) implies that only p-values below 0.0004 should be considered statistically significant (see also the legend to Table S1). The assessment of the effects of the four stratification variables should be considered explorative. Seventh, we were unable to assess the impact of scientific sub-discipline on the results since the free text field (survey item A.1, Appendix S2) yielded imprecise data with large variation.

Data on non-publication rates in LAR are scarce. Sena et al, using the statistical “trim and fill” technique on a large number of animal experiments on acute ischemic stroke, estimated the non-publication rate to be 13.6 percent which was associated with a 30% overstatement of efficacy. [6] Evidence from clinical research on humans suggests that between 46 and 67 percent of studies are not published [14]–[17], and that in those published, positive findings are over-emphasized. [18], [19] The emergence of trial registration, and the joint statement of the International Committee of Medical Journal Editors on publication of randomized trials being conditional on a trial having a public trial registration number may have reduced these numbers. [20] We agree with Sena et al who argued that “non-publication is unethical since it deprives researchers of the accurate data they need to estimate the potential of novel therapies in clinical trials, but also because the included animals are wasted because they do not contribute to accumulating knowledge. In addition, research syntheses that overstate effects may lead to further unnecessary animal experiments testing poorly founded hypotheses.” [6].

Measures against the suppression of “negative” results can be categorized from the source, via upstream to more downstream measures. Since, in The Netherlands, all experiments must pass a Institutional Animal Care and Use Committee (IACUC) for ethics approval, IACUCs may play a crucial role in the registration of all LAR and prevention of publication bias. A system ensuring periodic follow-up of each experiment’s fate would reinforce such registration. It may be challenging to build a watertight system that simultaneously minimizes bureaucracy. Application of modern information technology may be crucial. One option to prevent that study results have an effect on the editorial decision is to initially submit manuscripts without any results. [21] Editors and peer reviewers would judge the importance of submissions through the background, hypotheses and methods sections. This would ensure that acceptance is not conditional on the results. More downstream measures include special journals, journal sections or repositories for “negative” results, such as the Journal of Negative Results in Biomedicine, The All Results Journals and Negative Results in Gynecological Oncology. [4], [22], [23] In addition, two journals, the Journal of Cerebral Blood Flow and Metabolism and Neurobiology of Aging, feature Negative Results sections with a very similar flavor. [24], [25] The Journal of Cerebral Blood Flow and Metabolism describes this section as follows: “Negative Results is intended to provide a forum for data that did not substantiate [.] a difference between the experimental groups, and/or did not reproduce published findings. Since the net effect of a Negative Result is to discourage repetition, the standards for acceptance as a Negative Result will be highly demanding. Typically, Type II error considerations are mandatory.” [24].

Statistical significance testing is probably a main driver of non-publication. This is especially unfortunate given the widespread errors involved in understanding the meaning of p-values.[26]–[28].

What are the implications for further research? As we have learnt from randomized trials in humans, follow-up of cohorts of study protocols may help us understand the magnitude and the causes of publication bias in LAR, which in turn may affect the research community’s motivation to deal with it. In the meantime, more research into statistical correction of publication bias seems useful.[29]–[31] Specifically, the comparison of various methods to deal with publication bias statistically, such as the trim and fill [32], regression-based methods [30], [33], [34], and capture-recapture [31] may be compared in simulation studies to assess their strengths and weaknesses in various situations.

## Supporting Information

Table S1 Statistically significant differences between medians are highlighted with an asterisk (*). An asterisk in the column that corresponds to the first level of one of the four variables (affiliation, size of animals, number of papers published, focus of experiments), refers to the difference between the medians of the first and second level. An asterisk in the second column refers to the difference between the second and third level. An asterisk in the third column refers to the difference between the first and the third level.
(DOCX)

Table S2 **All numbers are medians after bootstrapping the analysis 200 times.** † Bootstrapped quantile regression on the median, simultaneously adjusting for all four stratification variables, that were modeled as dummy variables. CI denotes confidence interval. The intercept of the fully adjusted model, that is, the estimate for the median proportion of papers published of not-for profit researchers working with small animals, having co-authored between 0 and 5 papers, and working on both fundamental and pre-clinical topics was 50 percent (95% 43.5–56.5).
(DOCX)

Table S3 **All numbers are medians after bootstrapping the analysis 200 times.** † Bootstrapped quantile regression on the median, simultaneously adjusting for all four stratification variables, that were modeled as dummy variables. CI denotes confidence interval. The intercept of the fully adjusted model, that is, the estimate for the median proportion of papers published of not-for profit researchers working with small animals, having co-authored between 6 and 20 papers, and working on both fundamental and pre-clinical topics was 80 percent (95% 73.9–86.1). § The group that (co-)authored 0–5 studies was excluded from this row because very junior investigators very often had either zero or 100 percent of their papers published.
(DOCX)

Appendix S1 (DOC)

Appendix S2 (PDF)
