# Supplementary material for: Publication Bias in Laboratory Animal Research: A Survey on Magnitude, Drivers, Consequences and Potential Solutions
Source: PLoS One. 2012 Sep 5;7(9):e43404. doi: 10.1371/journal.pone.0043404 (PMC3434185; doi:10.1371/journal.pone.0043404)
Supplement: Table S1 — Statistically significant differences between medians are highlighted with an asterisk (*). An asterisk in the column that corresponds to the first level of one of the four variables (affiliation, size of animals, number of papers published, focus of experiments), refers to the difference between the medians of the first and second level. An asterisk in the second column refers to the difference between the second and third level. An asterisk in the third column refers to the difference between the first and the third level. (DOCX) [file pone.0043404.s001.docx]

**Table S1. Breakdown of survey responses by four background characteristics**

**(numbers are medians (interquartile range))**

|  | **Q2**^§^**. Affiliation¶** | | **Q3. Size of animals†** | | | **Q4. Number of papers published†** | | | **Q5. Focus of experiments†** | | |  |
| --- | --- | --- | --- | --- | --- | --- | --- | --- | --- | --- | --- | --- |
| ***Question^$^***  ***Label^[Scale]$$^*** | ***Not-for-profit***  ***(n=421)*** | ***For-profit***  ***(n=21)*** | ***Small***  ***only***  ***(n=365)*** | ***Large***  ***only***  ***(n=57)*** | ***Both***  ***(n=32)*** | ***0-5***  ***(n=233)*** | ***6-20***  ***(n=120)*** | ***20+***  ***(n=101)*** | ***Fundamental***  ***only***  ***(n=108)*** | ***Both***  ***(n=302)*** | ***Pre-clinical***  ***only***  ***(n=44)*** | ***Total***  ***(n=454)*** |
| **Q6**  **% overall^a^** | 50***  (35-70) | 10  (5-50) | 50  (30-70) | 55  (40-70) | 67.5  (40-80) | 50**  (30-65) | 60  (36.3-75) | 60**  (40-70) | 60  (40-74.3) | 50  (32.3-70) | 50  (21.3-75) | ***50***  ***(32-70)*** |
| **Q7**^§§^  **% own work^a^** | 80***  (60-90) | 10  (5-38.8) | 75**  (50-90) | 90  (78.8-100) | 82  (50-96.3) | NA | 80  (50-90) | 80  (60-90) | 80  (55-95) | 80  (60-90) | 77.5  (12.5-92.3) | ***80***  ***(60-90)*** |
| **Q8**  **problem^b^** | 7  (5-8) | 7  (5-8) | 7  (5-8) | 7  (4-8) | 6.5  (3.3-8) | 7  (5-8) | 7  (5-8) | 7**  (4-8) | 7  (5-8) | 7  (5-8) | 7  (5-8) | ***7***  ***(5-8)*** |
| **Q9.1**  **P>5%^c^** | 4  (4-5) | 4  (4-5) | 4  (4-5) | 4*  (4-5) | 4***  (3-4) | 4  (4-5) | 4  (4-5) | 4  (4-5) | 4  (4-5) | 4  (4-5) | 4  (4-5) | ***4***  ***(4-5)*** |
| **Q9.2**  **instrum problems^c^** | 4*  (3-4) | 3  (2-4) | 4  (3-4) | 4  (3-4) | 4  (2-4) | 4  (3-4) | 3  (3-4) | 4  (3-4) | 3**  (3-4) | 4  (3-4) | 4  (3-4) | ***4***  ***(3-4)*** |
| **Q9.3**  **lack of time^c^** | 2***  (2-3) | 4  (3-4) | 2**  (2-3) | 3  (2-4) | 2  (1-4) | 2  (2-3) | 3  (2-4) | 2  (2-3) | 2  (2-4) | 2  (2-3) | 2  (2-4) | ***2***  ***(2-3)*** |
| **Q9.4**  **loss of interest^c^** | 2**  (2-3) | 3  (3-4) | 2  (2-4) | 3  (2-3) | 3  (2-3) | 2  (2-3) | 3  (2-4) | 3  (2-3) | 2  (2-3) | 3  (2-3) | 3  (2-4) | ***2.5***  ***(2-3)*** |
| **Q9.5**  **pilot study only^c^** | 3  (3-4) | 3  (2-4) | 3 (3-4) | 3  (2-4) | 3  (2-4) | 3  (3-4) | 3  (2-4) | 3  (3-4) | 4  (2-4) | 3  (3-4) | 3  (2-4) | ***3***  ***(3-4)*** |
| **Q10.1**  **senior/supervisor^d^** | 4  (3-5) | 4  (3-5) | 4  (3-5) | 4  (3-5) | 4  (3-5) | 4  (3-5) | 4  (3-5) | 4  (2.8-5) | 4  (3-5) | 4  (3-5) | 4  (3-5) | ***4***  ***(3-5)*** |
| **Q10.2**  **fellow/PhD^d^** | 3  (2-4) | 4  (2.5-4.5) | 3  (2-4) | 3  (2-4) | 3  (2-4) | 3  (2-4) | 3  (2-4) | 4  (2-4) | 3  (2-4) | 3  (2-4) | 4  (2-4) | ***3***  ***(2-4)*** |
| **Q10.3**  **editors^d^** | 4*  (3-5) | 3  (2-4) | 4  (3-4) | 4  (3-5) | 4  (3-5) | 4  (3-4.8) | 4  (3-4) | 4  (2-5) | 3  (3-4) | 4  (3-5) | 4  (3-4) | ***4***  ***(3-4)*** |
| **Q10.4**  **reviewer/referee^d^** | 4***  (3-5) | 3  (2-4) | 4  (3-4.3) | 4  (3-5) | 4  (3-5) | 4  (3-4) | 4  (3-5) | 4  (3-4.3) | 4  (3-5) | 4  (3-4) | 3  (2-4) | ***4***  ***(3-5)*** |
| **Q10.5**  **funders^d^** | 2*  (1-4) | 4  (1.5-5) | 2  (1-4) | 3  (2-4) | 2  (1-3) | 3  (1-4) | 3  (1-4) | 2*  (1-3) | 2  (1-4) | 2  (1-4) | 3  (1-4) | ***2***  ***(1-4)*** |
| **Q11.1**  **duplication^e^** | 8*  (7-9) | 9  (7-10) | 8  (7-9) | 8  (7-9) | 7  (5.8-9) | 8  (7-9) | 8  (7-9) | 8  (7-9) | 8  (7-9) | 8  (7-9) | 8.5  (7-10) | ***8***  ***(7-9)*** |
|  | **Q2**^§^**. Affiliation¶** | | **Q3. Size of animals†** | | | **Q4. Number of papers published†** | | | **Q5. Focus of experiments†** | | |  |
| **Q11.2**  **SR/MA bias^e^** | 8  (6-9) | 8  (7-9.5) | 8  (6-9) | 8  (7-9) | 7.5  (6-9) | 8  (7-9) | 8  (6-9) | 8  (6-9) | 8  (6-8) | 8  (7-9) | 8  (6-9) | ***8***  ***(7-9)*** |
| **Q11.3**  **initiation phase-1^e^** | 7  (6-9) | 6  (4.5-8) | 7  (6-9) | 7*  (6-8) | 5.5**  (5-7) | 7  (6-9) | 7  (5.3-8) | 7  (5-8.3) | 7**  (5-8) | 7  (6-9) | 7  (5-8) | ***7***  ***(6-8)*** |
| **Q12**  **easier to publish^f^** | 2  (2-3) | 2  (2-3) | 2  (2-3) | 2.5  (2-3) | 2.5  (2-3) | 3  (2-3) | 2.5  (2-3) | 2  (2-3) | 2  (2-3) | 2  (2-3) | 2.5  (2-3) | ***2***  ***(2-3)*** |
| **Q14.1**  **duplication^g^** | 4  (3-4) | 4  (3-4) | 4  (3-4) | 4  (3-4) | 4  (3-4) | 4  (3-4) | 4  (3-4) | 3  (3-4) | 4  (3-4) | 4  (3-4) | 4  (3-4) | ***4***  ***(3-4)*** |
| **Q14.2**  **validity of SRs^g^** | 2  (2-3) | 2  (2-3) | 2  (2-3) | 2  (2-3) | 3  (2-3) | 2  (2-3) | 2  (2-3) | 2  (2-3) | 2  (2-3) | 2  (2-3) | 2  (2-3) | ***2***  ***(2-3)*** |
| **Q14.3**  **competition^g^** | 3*  (3-4) | 4  (3-4) | 3  (3-4) | 3  (3-4) | 3  (3-4) | 3  (3-4) | 3  (3-4) | 3  (3-4) | 3  (3-4) | 3  (3-4) | 3  (3-4) | ***3***  ***(3-4)*** |
| **Q14.4**  **bureaucracy^g^** | 2*  (1-3) | 2  (2-3) | 2  (1-3) | 2  (1-3) | 2  (1.5-2.5) | 2  (1-3) | 2  (1-2) | 2  (1-2) | 2  (1-3) | 2*  (1-2) | 2  (2-3) | ***2***  ***(1-3)*** |
| **Q14.5**  **scientif progress^g^** | 2  (2-3) | 2  (2-3) | 2  (2-3) | 2  (2-3) | 3  (2-3) | 2  (2-3) | 2.5  (2-3) | 3*  (2-3) | 2  (2-3) | 2  (2-3) | 2  (2-3) | ***2***  ***(2-3)*** |
| **Q15.1**  **duplication^g^** | 4*  (3-4) | 4  (4-4) | 4  (3-4) | 4  (3-4) | 4  (3-4) | 4  (3-4) | 4  (3-4) | 4  (3-4) | 4  (3-4) | 4  (3-4) | 4  (4-4) | ***4***  ***(3-4)*** |
| **Q15.2**  **validity of SRs^g^** | 2*  (2-3) | 2  (2-2) | 2  (2-3) | 2  (2-3) | 2  (2-2.8) | 2  (2-3) | 2  (2-3) | 2  (2-3) | 2  (2-3) | 2  (2-3) | 2  (2-2) | ***2***  ***(2-3)*** |
| **Q15.3**  **competition^g^** | 3  (3-4) | 4  (3-4) | 3  (3-4) | 3  (3-4) | 3  (3-4) | 3  (3-4) | 3  (3-4) | 3  (3-4) | 3  (3-4) | 3  (3-4) | 3  (3-4) | ***3***  ***(3-4)*** |
| **Q15.4**  **bureaucracy^g^** | 2*  (2-3) | 2  (2-3) | 2  (1-3) | 2  (2-3) | 2  (2-3) | 2  (2-3) | 2  (2-3) | 2*  (1-2) | 2  (1-3) | 2*  (2-3) | 2  (2-3) | ***2***  ***(2-3)*** |
| **Q15.5**  **scientif progress^g^** | 2  (2-3) | 2  (2-3) | 2  (2-3) | 2  (2-3) | 2  (2-3) | 2  (2-3) | 2  (2-3) | 2**  (2-3) | 2  (2-3) | 2  (2-3) | 2  (2-3) | ***2***  ***(2-3)*** |

Statistically significant differences between medians are highlighted with an asterisk (*). An asterisk in the column that corresponds to the first level of one of the four variables (affiliation, size of animals, number of papers published, focus of experiments), refers to the difference between the medians of the first and second level. An asterisk in the second column refers to the difference between the second and third level. An asterisk in the third column refers to the difference between the first and the third level.

* = 0.01≤p<0.05

** = 0.001≤p<0.01

*** = p<0.001

NA = not applicable

¶: Differences between the two columns were tested using the Mann-Whitney U test.

†: Differences between the two columns were tested using the Kruskal-Wallis test and Tukey’s post hoc test.

Note that we performed 121 statistical significance tests, namely, (27 + (3 x 27) tests plus an additional 13 post-hoc tests. We applied a Bonferroni correction for multiple testing, and consider only p-values < 0.05/121 = 0.00041322 (or 0.04%) as statistically significant.

§: Respondents that had both for-profit and not-for-profit affiliations was excluded from these categories.

§§: The group that (co-)authored 0-5 studies was excluded from this row because very junior investigators very often had either zero or 100 percent of their papers published.

$$: Scale:

a = 0-100%

b = 1-10 (not at all problematic (1) - extremely problematic (10))

c = 1-5 (totally unimportant (1) - very important (5))

d = 1-5 (least important (1) - most important (5))

e = 1-10 (totally unimportant (1) - extremely important (10))

f = 1-3 (1=never, 2=sometimes, 3=often/always)

g = 1-5 (extreme increase (1) - extreme decrease (5))

$: Question:

*Q1: What is your main field of expertise? (Omitted)*

Q2: What are your affiliations?

Q3: What is the size of the animals you spent most of your time working with?

Q4: How many peer-reviewed animal research papers have you (co-)authored?

Q5: Do your experiments focus more on fundamental or on pre-clinical topics?

Q6: Overall, what percentage of ethics-approved experiments performed in experimental animal research do you think is published?

Q7: Overall, what percentage of animal experiments you have been involved in have been published on (at least one publication)?

Q8: Do you consider publication bias a problem for experimental animal research? *(1=not at all problematic, 10=extremely problematic)*

Q9: According to you, what are important causes of non-publication in experimental animal research? *(1=totally unimportant, 5=very important)*

1. Lack of statistically significant differences (“negative” findings)

2. Instrumentation/technical problems

3. Lack of time to write manuscripts

4. Loss of interest

5. Many studies are seen as pilot studies only

Q10: Who are responsible for non-publication in experimental animal research? *(1=least important, 5=most important)*

1. Researchers (senior/supervisor)

2. Researchers (research fellow/PhD)

3. Editors

4. Reviewers/Referees

5. Funders

Q11: Do you think that publication bias is important for experimental animal research with respect to:… *(1=totally unimportant, 10=extremely important)*

1. Duplication of research efforts

2. Bias in literature reviews or meta-analyses

3. Initiation of phase-1 clinical trials in humans

Q12: If there were initiatives to make the publishing of negative results or comments on why an experiment could not be completed much easier, for example an (anonymous) online database or (online) journals of negative results, would you use them? *(1=never/sometimes, 2=often, 3=always)*

*Q13: If never, why not? NOT RELEVANT FOR THIS TABLE*

Q14: Mandatory anonymous publication of research protocols of all ethics-approved animal research experiments in a publically available database would change:… *(1=extreme increase, 5=extreme decrease)*

1. Duplication of research efforts

2. Validity of literature reviews

3. Certainty that competing investigators do not catch up

4. Bureaucracy

5. Overall scientific progress

Q15: Mandatory anonymous publication of a brief structured form in a publically available database, that gave main results or explained why an experiment could not be completed would change:… *(1=extreme increase, 5=extreme decrease)*

1. Duplication of research efforts

2. Validity of literature reviews

3. Certainty that competing investigators do not catch up

4. Bureaucracy

5. Overall scientific progress
