# Supplementary material for: Publication Bias in Laboratory Animal Research: A Survey on Magnitude, Drivers, Consequences and Potential Solutions
Source: PLoS One. 2012 Sep 5;7(9):e43404. doi: 10.1371/journal.pone.0043404 (PMC3434185; doi:10.1371/journal.pone.0043404)
Supplement: Table S3 — All numbers are medians after bootstrapping the analysis 200 times. † Bootstrapped quantile regression on the median, simultaneously adjusting for all four stratification variables, that were modeled as dummy variables. CI denotes confidence interval. The intercept of the fully adjusted model, that is, the estimate for the median proportion of papers published of not-for profit researchers working with small animals, having co-authored between 6 and 20 papers, and working on both fundamental and pre-clinical topics was 80 percent (95% 73.9–86.1). § The group that (co-)authored 0–5 studies was excluded from this row because very junior investigators very often had either zero or 100 percent of their papers published. (DOCX) [file pone.0043404.s003.docx]

Table S3. Regression analyses showing the multivariably adjusted effect of the four stratification variables on the estimate of the percentage of ethics-approved animal experiments that respondents had been personally involved in and that have been published on (N=210)^§^.

| **Stratification variable** | **Crude** | **95% CI** | **P-value** | **adjusted^†^** | **Adjusted 95% CI** | **P-value** |
| --- | --- | --- | --- | --- | --- | --- |
| Affiliation |  |  |  |  |  |  |
| Not-for profit (reference) |  |  |  |  |  |  |
| For-profit | -65 | -87.6 – -42.4 | <0.000 | -65 | -89.7 – -40.3 | <0.000 |
|  |  |  |  |  |  |  |
| Animal size |  |  |  |  |  |  |
| Small (reference) |  |  |  |  |  |  |
| Large | -5 | -22.0 – 12.0 | 0.562 | 10 | 1.1 – 18.9 | 0.028 |
| Large and small | 10 | -7.5 – 27.5 | 0.261 | 0 | -16.5 – 16.5 | 1.000 |
|  |  |  |  |  |  |  |
| Number of co-authored papers |  |  |  |  |  |  |
| 6-20 (reference) |  |  |  |  |  |  |
| At least 21 | 0 | -6.7 – 6.7 | 1.000 | 0 | -6.7 – 6.7 | 1.000 |
|  |  |  |  |  |  |  |
| Focus of experiments |  |  |  |  |  |  |
| Fundamental and pre-clinical (reference) |  |  |  |  |  |  |
| Fundamental only | 0 | -7.5 – 7.5 | 1.000 | 0 | -9.4 – 9.4 | 1.000 |
| Preclinical only | -5 | -44.1 – 34.1 | 0.801 | -5 | -28.1 – 18.1 | 0.670 |

All numbers are medians after bootstrapping the analysis 200 times. † Bootstrapped quantile regression on the median, simultaneously adjusting for all four stratification variables, that were modeled as dummy variables. CI denotes confidence interval. The intercept of the fully adjusted model, that is, the estimate for the median proportion of papers published of not-for profit researchers working with small animals, having co-authored between 6 and 20 papers, and working on both fundamental and pre-clinical topics was 80 percent (95% 73.9 – 86.1). § The group that (co-)authored 0-5 studies was excluded from this row because very junior investigators very often had either zero or 100 percent of their papers published.
