# Supplementary material for: Publication Bias in Laboratory Animal Research: A Survey on Magnitude, Drivers, Consequences and Potential Solutions
Source: PLoS One. 2012 Sep 5;7(9):e43404. doi: 10.1371/journal.pone.0043404 (PMC3434185; doi:10.1371/journal.pone.0043404)
Supplement: Appendix S1 — (DOC) [file pone.0043404.s004.doc]

Dear colleague,
Recently, I may have contacted you or one of your colleagues by phone. I announced a survey on "Publication Bias in animal studies." In this e-mail you will find the link to the (electronic) survey.

[web link was put here]

The survey is short, online and contains 15 questions (and two optional fields). Completion of the survey should not take more than about 10 minutes.

The Professional Association of Animal Welfare Officers facilitates distributing the survey. I kindly urge you to distribute this link to all animal researchers in your institute. For your convenience, you find on the next page an introductory text that you may copy-paste into your e-mail to the animal researchers, perhaps after some adjustment.

The research team would appreciate it if you include junior animal researchers since a possible contrast of opinions associated with seniority may be interesting.

I’d appreciate it if you’d report back to me to how many people you sent the survey. In that way, we can calculate the response rate. If you know a certain percentage of the addresses from your file is no longer current, I’d appreciate an estimate of this percentage.

For any questions regarding the survey, please contact Gerben ter Riet (020 5664640, Email g.terriet @ amc.nl).

Yours sincerely, on behalf of the research team


Dr. Gerben ter Riet, physician-epidemiologist

Dear colleague,

Research with “negative” results is not always published. As a consequence new research may be started although elsewhere it may be known that the new research is a dead end street. This practice wastes time and resources. In addition, literature reviews are not always reliable if positive findings are over-represented. In clinical research it is known that roughly half is never fully published. Lack of statistical significance likely plays an important role. This phenomenon is called *publication bias*. Little is known about publication bias in laboratory animal research.

A research team of the Academic Medical Center (AMC) in Amsterdam asks for your cooperation to a short survey into publication bias in your field.

The survey is short, internet-based and contains 15 questions (and two optional fields). Completion of the survey should not take more than about 10 minutes. Your anonymity and that of your institute are guaranteed.

The link to the survey is:

[web link was put here]

I sincerely hope that you will participate.

Kind regards,

[local animal welfare officer’s name]
