# Supplementary material for: Publication Bias in Laboratory Animal Research: A Survey on Magnitude, Drivers, Consequences and Potential Solutions
Source: PLoS One. 2012 Sep 5;7(9):e43404. doi: 10.1371/journal.pone.0043404 (PMC3434185; doi:10.1371/journal.pone.0043404)
Supplement: Appendix S2 — (PDF) [file pone.0043404.s005.pdf]

Survey on publication bias in experimental animal research

Survey on publication bias in experimental animal research

Dear Colleague,

In clinical research, about half of the ethics-committee-approved projects are published. Lack of statistical significance appears to be the major reason for not publishing the outcomes of studies. The consequence is an excess of “positive” results in the literature. This selective publishing or “publication bias” distorts scientific evidence and negatively affects the validity of, for example, literature reviews and meta-analyses.

In experimental animal research, work on publication bias is scarce. Through this short survey, we'd like to explore what animal researchers think about publication bias. This survey is short. It will take less than 10 minutes. We would very much appreciate your participation.

Data will be analyzed with strict adherence to anonymity, both at the level of persons and institutes. In particular, the investigators will not have access to respondents' IP-addresses. We intend to submit a manuscript describing the results of this survey to a scientific journal.

Thank you very much for your consideration,

Gerben ter Riet, MD, PhD, Dept of General Practice, AMC, UvA,
Lotty Hooft, PhD, Dutch Cochrane Centre, AMC, UvA
Daniel Korevaar, Research Fellow, Dept of General Practice, AMC, UvA
Ronald Oude Elferink, Prof, MD, PhD, Tytgat Institute for Liver and Intestinal Research, UVA
Peter Sterk, MD, PhD, Dept Respiratory Medicine, AMC, UVA
René Lutter, PhD, Dept of Pulmonology, AMC, UvA
Ron van Noorden, Prof, MD, Dept of Cell Biology and Histology, AMC, UVA

E-mail: g.terriet@amc.nl

Survey on publication bias in experimental animal research

PART A: Background data.

What is your main field of expertise?

Survey on publication bias in experimental animal research

PART A: Background data.

What are your affiliations?
(Tick more than one box if appropriate.)

- University
- Not-for-profit institute
- For-profit company
- Other

Survey on publication bias in experimental animal research

PART A: Background data.

What is the size of the animals you spent most of your time working with? (Tick one box.)

- Small (e.g. birds, chicken, rodents, fish, amphibians, reptiles)
- Large (other)
- Both

Survey on publication bias in experimental animal research

PART A: Background data.

How many peer-reviewed animal research papers have you (co-)authored? (Tick one box.)

- 0
- 1-5
- 6-20
- 21-50
- 51-100
- 100+

Survey on publication bias in experimental animal research

PART A: Background data.

Do your experiments focus more on fundamental or on pre-clinical topics? (We define pre-clinical as animal research to find out if a drug, procedure or treatment is likely to be useful in humans. All other research is defined as fundamental.)
(Tick one box.)

- Fundamental only
- More fundamental
- About equal
- More pre-clinical
- Pre-clinical only

PART B: Your opinion on the extent and nature of publication bias in your f...

Overall, what percentage of ethics-approved experiments performed in experimental animal research do you think is published?

PART B: Your opinion on the extent and nature of publication bias in your f...

Overall, what percentage of animal experiments you have been involved in have been published on (at least one publication)?

PART B: Your opinion on the extent and nature of publication bias in your f...

Do you consider publication bias a problem for experimental animal research? (Tick one box on a 10 point scale.)

	not at all problematic									extremely problematic
Publication bias is:	☐	☐	☐	☐	☐	☐	☐	☐	☐	☐

PART B: Your opinion on the extent and nature of publication bias in your f...

According to you, what are important causes of non-publication in experimental animal research?
(Tick one box per item.)

	totally unimportant	unimportant	neutral	important	very important
Lack of statistically significant differences (“negative” findings)					
Instrumentation/technical problems					
Lack of time to write manuscripts					
Loss of interest					
Many studies are seen as pilot studies only					

Other (please specify)

PART B: Your opinion on the extent and nature of publication bias in your f...

Who are responsible for non-publication in experimental animal research?
(Please assign a rank number between 1 and 5, where 1 indicates “most important” and 5 “least important”. If you think that two or more parties are equally important, give them the same rank.)

	1 (most important)	2	3	4	5 (least important)
Researchers (senior/supervisor)					
Researchers (research fellow/PhD)					
Editors					
Reviewers/Referees					
Funders					

Do you think that publication bias is important for experimental animal research with respect to:
(Tick one box per item.)

	totally unimportant									extremely important
Duplication of research efforts										
Bias in literature reviews or meta-analyses										
Initiation of phase-1 clinical trials in humans based on biased cumulative evidence										

PART C: Your opinion on some solutions that were suggested to fight publica...

If there were initiatives to make the publishing of negative results or comments on why an experiment could not be completed much easier, for example an (anonymous) online database or (online) journals of negative results, would you use them?
(Tick one box.)

- ☐ Never
- ☐ Sometimes
- ☐ Often
- ☐ Always

PART C: Your opinion on some solutions that were suggested to fight publica...

If never, why not?

(Tick more than one box if appropriate.)

- Lack of time
- Making sure that competing investigators do not catch up
- Not interested
- Do not see the point
- Other (please specify)

Survey on publication bias in experimental animal research

PART C: Your opinion on some solutions that were suggested to fight publica...

In theory, publication bias could be controlled if Institutional Animal Care and Use Committees (Animal Ethics Committees) registered all research protocols, monitored projects' progress, and were authorized to demand a brief structured form, that gave main results or explained why an experiment could not be completed. These forms, linked to their protocol, would then be published anonymously.

Mandatory anonymous publication of research protocols of all ethics-approved animal research experiments in a publically available database would change (levels of): (Tick one box per item.)

	extreme increase	increase	hardly any change	decrease	extreme decrease
Duplication of research efforts					
Validity of literature reviews and meta-analyses of experimental animal research					
Certainty that competing investigators do not catch up					
Bureaucracy					
Overall scientific progress					

Survey on publication bias in experimental animal research

PART B: Your opinion on the extent and nature of publication bias in your f...

Mandatory anonymous publication of a brief structured form in a publically available database, that gave main results or explained why an experiment could not be completed would change (levels of): (Tick one box per item.)

	extreme increase	increase	hardly any change	decrease	extreme decrease
Duplication of research efforts					
Validity of literature reviews and meta-analyses of experimental animal research					
Certainty that competing investigators do not catch up					
Bureaucracy					
Overall scientific progress					

Survey on publication bias in experimental animal research

You have now completed the survey.

Please press "done" to complete the survey.

THANK YOU VERY MUCH FOR YOUR PARTICIPATION !

Please use the box below to express any thoughts or comments on this survey.

I would like to receive a summary of the results of this survey. My email address is:
